# Phylogenetic Group Determination of *Escherichia coli* Isolated from Animals Samples

**DOI:** 10.1155/2015/258424

**Published:** 2015-09-02

**Authors:** Fernanda Morcatti Coura, Soraia de Araújo Diniz, Marcos Xavier Silva, Jamili Maria Suhet Mussi, Silvia Minharro Barbosa, Andrey Pereira Lage, Marcos Bryan Heinemann

**Affiliations:** ^1^Departamento de Medicina Veterinária Preventiva, Escola de Veterinária, Universidade Federal de Minas Gerais (UFMG), Avenida Antônio Carlos 6627, 30123-970 Belo Horizonte, MG, Brazil; ^2^Escola de Medicina Veterinária e Zootecnia, Universidade Federal do Tocantins (UFT), Inspeção e Tecnologia de Carnes e Derivados, BR 153, Km 112, 77804-970 Araguaína, TO, Brazil; ^3^Departamento de Medicina Veterinária Preventiva e Saúde Animal, Faculdade de Medicina Veterinária e Zootecnia, Universidade de São Paulo, Avenida Professor Orlando Marques de Paiva 87, 05508-270 São Paulo, SP, Brazil

## Abstract

This study analyzes the occurrence and distribution of phylogenetic groups of 391 strains of *Escherichia coli *isolated from poultry, cattle, and water buffalo. The frequency of the phylogroups was A = 19%, B1 = 57%, B2 = 2.3%, C = 4.6%, D = 2.8%, E = 11%, and F = 3.3%. Phylogroups A (*P* < 0.001) and F (*P* = 0.018) were associated with *E. coli *strains isolated from poultry, phylogroups B1 (*P* < 0.001) and E (*P* = 0.002) were associated with *E. coli *isolated from cattle, and phylogroups B2 (*P* = 0.003) and D (*P* = 0.017) were associated with *E. coli *isolated from water buffalo. This report demonstrated that some phylogroups are associated with the host analyzed and the results provide knowledge of the phylogenetic composition of *E. coli* from domestic animals.

## 1. Introduction

*Escherichia coli* is a gram-negative, fermentative, rod-shaped bacterium that is the major facultative anaerobic bacterium in the intestinal tract of most animal species.* E. coli* cause enteric and extraintestinal diseases in animals [1]. Avian pathogenic* E. coli* (APEC) are associated mainly with extraintestinal infections and with cellulitis [2]. EHEC (Enterohemorrhagic* Escherichia coli*), especially O157:H7, cause hemorrhagic colitis and hemolytic uremic syndrome in humans. Contaminated foods of animal origin are the main form of transmission of EHEC to humans. Besides raw milk, beef and broiler carcasses are an important source of EHEC [3].

Differentiation of pathogenic strains from normal microbiota is based on the production of virulence factors and on the identification of mechanisms by which they cause disease, which allow their classification into pathotypes [1]. Combinations of genes can be used to cluster* E. coli* strains into phylogenetic groups. Multilocus sequence typing (MLST) data improves the understanding of* E. coli* phylogenetic structure and allowed strains to be classified in one of the seven phylogroups A, B1, B2, C, D, E, and F [4]. MLST is the best technique for typing* E. coli* but the sequence type (ST) provided in the analysis does not directly allow classification into phylogroups. Thus, it is necessary to determine the correspondence between ST and phylogroups, with the latter performed by means of the “Clermont method” [4, 5].

The understanding of* E. coli* structure showed that the strains belonging to the different phylogroups are not dispersed randomly and are associated with the source of isolation [4]. Phylogenetic studies are important to improve the understanding of* E. coli* population and the relationship of strains and their hosts and disease [6]. Therefore, this study aims to analyze the occurrence and distribution of phylogenetic groups of* Escherichia coli* isolated from different domestic animals.

## 2. Materials and Methods

Seventy-one fecal specimens were collected from water buffalo calves up to 90 days old from five farms located in Minas Gerais State, Brazil. All farms bred only Mediterranean and/or Murrah buffalos for milk production. Also, one hundred seventy-one fecal samples were collected from crossbred Holstein-Gyr calves up to 90 days old born in a dairy herd located in the city of Martinho Campos, Minas Gerais State, Brazil. One gram of feces of each animal was diluted in 3.0 mL of PBS pH 7.2. From that suspension, 1.0 mL was inoculated into 9.0 mL of buffered peptone water and incubated for 18–24 h at 37°C. After incubation, an aliquot of the preenriched cultures was plated onto MacConkey agar plate and incubated at 37°C for 18–24 h. Three* E. coli* colonies were identified biochemically and collected from each agar plate [7].

One hundred forty-nine poultry carcasses were collected from one slaughterhouse under Federal Sanitary Inspection localized in Tocantins State, Brazil, which slaughter broiler chickens from São Paulo, Tocantins, Goiás, and the Federal District. Swabs of the air bags and trachea carcasses (respiratory tract), liver, and heart were collected. The swabs of each organ were placed in a sterile test tube containing 0.9 mL of 0.85% saline and refrigerated until processing. The swabs were processed individually [7] for isolation and identification of* E*.* coli*. Briefly, swabs were streaked onto MacConkey Agar and incubated at 37°C for 24 hours. Then, after checking the growth of colonies on MacConkey Agar, up to three lactose positive colonies were characterized biochemically [7].

Only one* E. coli* isolated from each host sample was used for further phylogenetic characterization and statistical analysis. The* E. coli* strains were tested for* chuA*,* yjaA*, TSPE4.C2,* arpA*, and* trpA* genes by PCR and this characterized phylogenetic groups A, B1, B2, C, D, E, and F [4]. Amplified DNA was resolved on a 2% agarose gel, stained with 0.5 *μ*g/mL of ethidium bromide and photographed under UV light.

The Shannon and Simpson diversity indexes were calculated [8]. All data analyses were carried out using the Stata/SE 12.0 software. The association between the host and phylogroups was studied using the chi-square test. The results were each expressed as *P* value. The result was considered to be significant at *P* ≤ 0.05. Correspondence analysis (CA) was used to compare the categories of host and phylogroup using the Stata/SE 12.0. In the CA analysis, the relationship between the categories is represented in a two-dimensional graph.

## 3. Results

A total of 391* E. coli* strains were analyzed in the study. These were assigned to one of the seven phylogenetic groups (Table 1). The frequency of phylogroups was the following: A = 19%, B1 = 57%, B2 = 2.3%, C = 4.6%, D = 2.8%, E = 11%, and F = 3.3%. The diversity indexes (Shannon and Simpson) are shown in Table 1.

A chi-squared test checked the association between the host and phylogenetic group. Identification of phylogroups A (*P* < 0.001) and F (*P* = 0.018) was associated with* E*.* coli* strains isolated from poultry, while phylogroups B1 (*P* < 0.001) and E (*P* = 0.002) were associated with* E. coli* strains isolated from cattle. Phylogroups B2 (*P* = 0.003) and D (*P* = 0.017) were associated with* E. coli* strains isolated from water buffalo.

**Figure 1:**
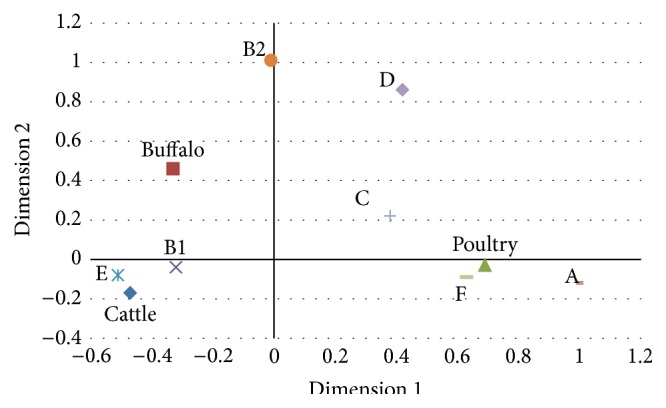
CA for the categories analyzed. Host and phylogroup that are similar fall close. This two-dimensional representation explains 100% of the total variation, with 85.20% explained by the 1st dimension and 14.80% by the 2nd dimension.

The CA was performed using the host and the phylogenetic group distribution. The bidimensional representation of phylogroup distribution in each of the three hosts is shown in Figure 1. The bidimensional representation explains 100% of the total variation with 85.20% explained by the 1st dimension and 14.80% by the 2nd dimension.

## 4. Discussion

This report determined the occurrence of phylogroup of* Escherichia coli* and demonstrated that some phylogroups are associated with the host analyzed. Phylogenetic studies help understand* E. coli* and its hosts and disease [6]. Moreover, food producing animals, such as cattle, water buffalo, and poultry, are an important source of EHEC in the food chain [9]. In Southern Brazil, beef and dairy cattle and water buffalos are important economically [10]. In addition, Brazil is the third largest producer of chicken meat and the largest exporter of this product [11].

The diversity indexes (Shannon and Simpson index) show that there is greater diversity in* E. coli* strains isolated from poultry than water buffalo and cattle (Table 1). The Shannon and Simpson index obtained for poultry were similar to those estimated by Carlos et al. [12], while cattle indices were lower than those obtained by Carlos et al. [12]. Cattle and water buffalo differ from poultry and share some characteristics such as diet and gut morphology; this may account for the differences in the diversity indexes.

Correspondence analysis examines the relationship between categorical nominal data using a contingency table of the categorical variables and transforms nonmetric data to metric data, allowing the mapping visualization, indicating that the higher the association is, the closer together the variables are in the maps [13]. Even though CA can be used to evaluate complex associations among variables, it is sometimes not sufficient to completely evaluate the associations of variables and it is suggested to use another simple (unconditional) analysis (e.g., chi-square) or multivariate analysis (e.g., logistic regression) in conjunction to complement the analytical procedures [14]. This is why we used both chi-square and CA to evaluate the relationship of* E. coli* phylogenetic group and the host from which the strains were isolated.

Our results indicate that B1 is the main phylogroup of* E. coli* isolated from domestic animals followed by phylogroup A. The results of the chi-square test and the CA agreed and showed that phylogroups B1 and E are associated with* E. coli* strains isolated from cattle and phylogroups A and F with poultry.* E. coli* strains from water buffalo were associated with phylogroups B2 and D in the chi-square but the CA showed no clear association, since these variables were not so close in the graph. Although CA did not indicate a strong association of* E. coli* strains of phylogroups B2 and D isolated from water buffalos, these two phylogroups were relatively closer to water buffalo than poultry and cattle and together with the chi-square results indicate that there is a tendency to detect* E. coli* strains of phylogroups B2 and D isolated from water buffalos.

The Extraintestinal pathogenic* E. coli* (ExPEC) strains are clustered mostly in groups B2 and D showing a link between phylogeny and virulence [15]. Our findings indicate that* E. coli* strains isolated from water buffalo calves may carry pathogenic characteristics of extraintestinal pathogenic* E. coli*. Studies have shown that phylogroup A is the most common phylogroup of strains obtained from ominivorous mammals and phylogroup B1 is prevalent in those isolated from herbivorous mammals [12, 16]. According to Gordon and Cowling [17], host habitat, diet, gut morphology, and body mass influence the distribution of the* E. coli* groups among the mammalian host. In the domestic animals analyzed here, diet and gut morphology seem to have influenced the distribution of the phylogroups. The CA can be used for molecular epidemiology studies to determine the phylogroup distribution among different hosts.

Molecular protocols such as sequencing the 16SrRNA gene and MLST are uneconomical for most screening purposes. Besides, MLST does not provide information concerning the phylogenetic group, and the phylogroup assignment depends on the “Clermont method” [5]. In our study, most phylogroups were detected in all three hosts studied; however, the chi-square test and the CA model indicate some host specificity. The PCR-based method to identify the seven phylogenetic groups was recently developed [4]; its use is rare in the literature. Our results provide knowledge of the phylogenetic composition of* E. coli* from domestic animals.

## Figures and Tables

**Table 1:** Distribution of the phylogenetic groups and diversity indexes among *E. coli *strains isolated from different domestic animals.

Host	Phylogenetic group								Diversity indexes	
	A	B1	B2	C	D	E	F	Total	Shannon	Simpson
Poultry	64	51	3	10	6	6	9	149	0.6230	0.6878
Cattle	8	127	1	4	0	28	3	171	0.3689	0.4185
Water buffalo	2	45	5	4	5	9	1	71	0.5416	0.5681
Total	74	223	9	18	11	43	13	391	—	—
